# Frequency of HBsAg variants in occult hepatitis B virus infected patients and detection by ARCHITECT HBsAg quantitative

**DOI:** 10.3389/fcimb.2024.1368473

**Published:** 2024-05-03

**Authors:** Chengshan He, Yang Liu, Xiudi Jiang, Zheng Xu, Zhouhong Xiang, Zhicheng Lu

**Affiliations:** Department of Clinical Laboratory, The Seventh People’s Hospital of Shanghai University of Traditional Chinese Medicine, Shanghai, China

**Keywords:** occult hepatitis B virus infection, S region mutation, major hydrophilic region, α determinant, missed detection

## Abstract

**Objective:**

To analyze the amino acid substitution caused by mutations in the major hydrophilic region (MHR) of the S-region genes in the serum samples of occult hepatitis B virus infection (OBI), and to explore the reasons for the missed detection of HBsAg.

**Method:**

The full-length gene of the S-region in hepatitis B virus(HBV) in the chronic hepatitis B virus(CHB)(10 samples) and OBI groups(42 samples) was amplified using a lab-developed, two-round PCR amplification technology. The PCR amplification products were sequenced/clone sequenced, and the nucleotide sequences of the S-region gene in HBV were compared to the respective genotype consensus sequence.

**Results:**

Only 20 of the 42 samples in the OBI group had the S-region genes successfully amplified, with the lowest HBV DNA load of 20.1IU/ml. As S-region genes in HBV, 68 cloned strains were sequenced. In the OBI and CHB groups MHR region, with a mutation rate of 3.21% (155/4828) and 0.70% (5/710). The genetic mutation rate was significantly higher in the OBI group than in the CHB group (P<0.05). The common mutation types in the MHR region were: I126T, L162R, K122E, C124R, and C147Y.Mutations at s122, s126, and s162 were associated with subgenotypes, most of which being C genotypes. The high-frequency mutation sites L162R and K122E found in this study have not been reported in previous literature.

**Conclusion:**

The results of this study confirmed that MHR mutations can cause the missed detection of HBsAg, giving rise to OBI.

## 1 Introduction

Infections from hepatitis B virus (HBV) have become an important global public health issue. The data released by the WHO in 2021 indicate that there are over 296 million people infected with chronic hepatitis B virus (CHB) worldwide (World Health Organization. Hepatitis B [EB/OL]., 2021), and about 650,000 people die each year from acute and chronic hepatitis, liver cirrhosis, hepatocellular carcinoma and other diseases caused by HBV infection. The problem of HBV infection is particularly severe in China. According to the materials released by the National Bureau of Disease Control and Prevention of the National Health Commission in 2013, around 500 million people in China were infected with HBV in 2006. With about 93 million carriers of hepatitis B surface antigen (HBsAg) (Bureau of Disease Control and Prevention. China has achieved remarkable results in controlling hepatitis B[EB/OL], 2013), China had the greatest population of HBsAg carriers in the world. HBsAg is an important serological marker for clinical diagnosis of HBV infection. Notably, there is a special form of HBV infection clinically, where the infected person tests negative for HBsAg in the blood, but positive for HBV DNA in the liver or blood. This is called occult hepatitis B infection (OBI) (Wang et al., 2020). The existence of OBI has brought great hidden dangers to the prevention and screening of clinical hepatitis B, the monitoring of treatment and the safety of blood transfusion. The mechanism of OBI is yet to be clarified. At present, the mainstream theory holds that the mechanism causing OBI mainly comprises the following aspects: genetic mutations in the HBV (Ahmadi Ghezeldasht et al., 2023), suppression of the host’s immune response (Raimondo et al., 2008), insufficient sensitivity of the HBsAg test kit (Sadeghi et al., 2017), and mixed infection involving other viruses (Chen et al., 2003). Among these aspects, mutations in the S genomic region of HBV is a hotspot of current researches, and is widely regarded as one of the primary reasons causing OBI (Ireland et al., 2000; Hsu and Yeh, 2011; Huang et al., 2012). Mutations in the “α” determinant (amino acid position aa124~147) in the major hydrophilic region (MHR) of S gene in HBV (amino acid position aa 99~169) are the most common. The “α” determinant is the immunogenic region of HBsAg (Huang et al., 2017), and its second loop structure is the major antigenic determinant of HBsAg (Zhang et al., 2017). Mutations of a single sequence site or multiple adjacent ones in this region can cause changes in the conformational epitope of HBsAg protein, reducing the antigenicity of HBsAg, thereby affecting the binding reaction of HBsAg with the corresponding antibody (Yan et al., 2022). In addition, genetic mutations in the MHR will also affect the expression and secretion of viral HBsAg, making it lower than the detection limit of clinical HBsAg test reagents or causing the viral load in the circulation to be at a low level, thereby leading to missed detection of HBsAg (Qi et al., 2023).

This study aims to identify which genetic mutations may affect the detection of HBsAg by analyzing the mutation sites in the MHR region of the S gene in OBI samples. The research results can help clarify the reasons for causing OBI, correctly explain the missed detection of HBsAg in clinical practice, and provide theoretical basis for establishing an effective serological detection method for HBsAg mutant strains and developing new diagnostic reagents.

## 2 Materials and methods

### 2.1 Sample collection

A total of 42 serum samples were collected from the outpatients and inpatients in Shanghai Seventh People’s Hospital who fit the criteria of the OBI experimental group. All samples were HBsAg non-reactive, HBcAb reactive, and HBcAb S/Co>7.5. The HBV DNA of the serum was above 20IU/ml detected by the real-time fluorescence quantitative PCR method (Perkin Elmer, Shanghai). The serum samples of ten CHB patients with a clinical history of more than three years were collected as the control group, and the HBV markers of their serum were HBsAg positive, HBeAg positive and HBcAb positive, and the HBV DNA were above 1.0×10^6^ IU/ml. The five serological markers of HBV were tested with Abbott i2000 detection system chemiluminescence immunoassay (CMIA), and the cut-off value of reaction were HBsAg>0.05IU/mL, Hepatitis B surface Antibody (HBsAb)>10mIU/mL, and Hepatitis B e Antigen (HBeAg)>1 S/Co, Hepatitis B e Antibody (HBeAb)<1 S/Co, HBcAb>1 S/Co. The samples of the experimental group were divided into the following five subgroups: only HBcAb reactive, HBsAg, HBsAb, HBeAg and HBeAb non-reactive (group A); HBsAb and HBcAb reactive, HBsAg, HBeAg and HBeAb non-reactive (group B); HBeAb and HBcAb reactive, HBsAg, HBsAb, and HBeAg non-reactive (group C); HBsAb, HBeAb and HBcAb reactive, HBsAg and HBeAg non-reactive (group D); HBsAb, HBeAg, and HBcAb reactive, HBsAg and HBeAb non-reactive (group E). A CHB control group (group F) was also established. All serum samples included in the experimental group and the control group were negative for HCV antibodies and HIV antibodies. This study was approved by the Medical Ethics Committee of our hospital (2018-IRBQYYS-020). The serum samples used in the research were all collected from serum samples discarded after clinical testing. The personal information of the patients was not disclosed, so informed consent was waived.

### 2.2 Gene Amplification in the S-region of HBV

We extracted DNA from the serum samples whose HBV DNA tested above 20 IU/ml using fluorescence quantitative PCR (Perkin Elmer, USA) with a nucleic acid magnetic bead isolation kit (Perkin Elmer, USA). A UV spectrophotometer (Nano Drop, Thermo Fisher Scientific, USA) was used to test the nucleic acid concentration (ng/μL). After the extraction was completed, the nucleic acid was divided into sterile non-enzyme-covered test tubes and stored in a -20°C refrigerator to avoid repeated freezing and thawing. The HBV genotype sequences that are prevalent in the Chinese population were downloaded from the NCBI website https://www.ncbi.nlm.nih.gov/nuccore/, and Primer5 was used to design two rounds of PCR primers P F: 5’-CCTKCTCGTGTTACAGGCGG-3’(187-206), R: 5’-CGRGCAACGGGGTAAAGG-3’(1140-1157), the amplified fragment was 971bp. K and R are degenerate bases, wherein K: G/T, R: A/G, The location of HBV S region gene base sequence was 157-837, and the amplification product lacks 31bp gene sequence about the front part of 157-187.The first-round and second-round primers were the same. DNA polymerase (Takara Premix Taq, Takara Biomedical, Beijing) was used for the PCR amplification of the target S-region genes in the HBV in a gradient PCR instrument (ABI Veriti Thermal Cycler, ABI, USA).First reaction: The reaction system consisted of 20 ng DNA template, 25μL Taq DNA polymerase premixture, 2μL primer, add DEPC water to 50μL. The reaction condition was predenaturation 98 °C for 30 s. Denatured 98°C 10 s, annealed 55°C 30 s and extended 72°C 65 s, cycling 30 times. The second round reaction: the first round PCR amplification product 1μL (1:10 dilution), primer 1μL, annealing at 62°C for 30 s, cycling 25 times, other conditions were the same as the first round reaction.

### 2.3 PCR product sequencing

The PCR products of the CHB control samples were directly sent for sequencing. The PCR products of the OBI samples were connected with a pMD18-T vector (Takara Biomedical, Beijing) and transformed into NcmDH5α competent cells (New Cell & Molecular Biotech, Suzhou) for cloning and sequencing. Five clones were selected from each sample for sequencing. The purified PCR products and TA cloned bacterial plasmids were sent to Suzhou Genewitz Biotechnology Co., Ltd. for sequencing using a sequencer (ABI3730, ABI, USA). We used the NCBI website (https://blast.ncbi.nlm.nih.gov/Blast.cgi) for nucleic acid sequence BLAST alignment to see if the genetic sequence came from the S-region of the HBV DNA. We used the website (www.ncbi.nlm.nih.gov/projects/genotyping/) to carry out the HBV genotyping of each sample, and used DNAMAN version 8.08.798 to compare and analyze the corresponding reference sequences of each genotype to obtain nucleic acid and amino acid mutation sequences. We were defined amino acid substitution resulting from base mutation as the standard for variant/mutation.

### 2.4 Statistical analysis

We used SPSS19.0 for data processing and statistical analysis. The group rates were compared using a χ2 test, and P<0.05 (two-sided) was considered statistically significant.

## 3 Results

### 3.1 Clinical information of the OBI patients

Among the 42 patients in the OBI experimental group, the HBV DNA load of 39 patients was below 200IU/ml, and the HBV DNA load of 25 patients was below 50IU/ml. There were more males than females among the 42 OBI patients, and most of them were middle-aged. Twelve infected patients had elevated serum alanine amino transferase(ALT), and three of them had both elevated ALT and elevated aspartate aminotransferase(AST) is shown in **Table 1**.

**Table 1:** Basic Clinical Information of the Patients with Positive HBV DNA.

Markers/group	total	A	B	C	D	E
N(%)	42	3(7.14%)	5(11.90%)	11(26.19%)	22(52.38%)	1(2.38%)
Gender(M/F)	27/15	3/0	2/3	7/4	15/7	0/1
Age(Median years)	42.5	41.1	45.9	36.3	40.5	39.8
ALT or AST>64U/L	12	0	2	3	6	1
HBV DNA load(IU/mL)						
20~100	29	1	3	9	15	1
100~200	10	2	2	2	4	0
>200	3	0	1	0	2	0
HBcAb titer(S/Co)						
7.5~9.0	19	2	3	5	9	0
9.1~9.9	19	1	2	5	10	1
≥10.0	4	0	0	1	3	0

Group A、B、C、D、E from Materials and Methods 1.Sample Collection part.

Group A: only HBcAb reactive. HBsAg, HBsAb, HBeAg and HBeAb non-reactive;

Group B: HBsAb and HBcAb reactive. HBsAg, HBeAg and HBeAb non-reactive;

Group C: HBeAb and HBcAb reactive. HBsAg, HBsAb, and HBeAg non-reactive;

Group D: HBsAb, HBeAb and HBcAb reactive, HBsAg and HBeAg non-reactive;

Group E: HBsAb, HBeAg, and HBcAb reactive, HBsAg and HBeAb non-reactive.

### 3.2 Results from the amplification and sequencing of the S-region genes in HBV

The 42 OBI samples were processed with the lab-developed two-round PCR reaction to amplify the S-region gene in HBV. Specific bands were successfully amplified in only 20 cases, of which twelve cases had a HBV DNA load below 10^2^IU/ml, and eight cases had a HBV DNA load below 30IU/ml, with a minimum load of 20.1IU/ml. The PCR products of the ten CHB control samples were electrophoresed and the target bands were clear. 20 OBI samples were cloned and sequenced, and five monoclonal strains were selected for each sample. A total of 100 clones were selected, of which 68 clones were recognized as S-region genes in HBV through BLAST comparison. The remaining 32 were considered other genomic components, primarily consisting of the human chromosome gene (PAX2) and pMD-18T empty plasmid, etc. The possible reason was that some non-specific bands were incorporated into the cutting of target gene bands, and the concentration of template DNA was too low, so the T-vector and template DNA could not be connected properly. All 20 OBI patient samples contained at least one clone with authentic S gene sequences. The purified PCR products of the ten cases in the CHB control group were directly sequenced. All samples were recognized as S-region genes in HBV through BLAST comparison. The 68 clones and ten CHB control samples were sequenced on the NCBI website for HBV genotyping, and three virus strains of different subtypes were obtained, namely B, C1, and C2. The serial numbers of the subtypes are as follows: Type B: AB602818, Type C1: AB014381, Type C2: M12906. DNAMAN was used to compare the sequenced S-region genes in HBV with their corresponding genotype reference sequences to determine the mutations at the base and amino acid sites. The specific information of the OBI and CHB group samples is shown in **Table 2**.

**Table 2:** Serological and molecular biological detection results of OBI and CHB groups.

Sample	HBV DNA (IU/mL)	Clone	Genotype	Mutations in MHR
A2	1.66E+02	Clone1,2,4	B,C1	K122E,I126T,T131A,M133L,C147Y,L162R,W163R
B1	1.73E+02	Clone2,4,5	B	T126A,S136T
B5	2.15E+02	Clone1	B	P127T,K141R
C5	1.69E+02	Clone1,2,4,5	C1	No
C9	2.75E+01	Clone1,2,3,4,5	B,C1	V106G,K122E,I126T,T140S,I152V,L162R/stop
C10	2.17E+01	Clone1,2,3,4,5	C1	V106G,L109P,S114P,K122E,C124R,I126T,A128V,I152V,L162R
D1	1.63E+02	Clone1,2,3,5	C1	Y100C,K122E,I126T,N146S,L162R
D2	1.42E+02	Clone2,3	B,C1	K122E,C124R,I126T,L162R
D5	5.29E+03	Clone1,4,5	C1	K122E,I126T,M133I,L162R
D6	2.28E+01	Clone1,3,5	B	I110V,C124R,I152T,W163R/stop
D7	3.67E+01	Clone2,3,4,5	B,C1	K122E,I126T,T148A,L162R
D8	5.36E+01	Clone1,2,4	B,C1	L109P,S114P,K122E,C124R,L162R
D10	3.05E+01	Clone1,2,3,4,5	C1	L109P,S114P,K122E,C124R,I126T,C138R,C139W,L162R
D13	2.86E+01	Clone1,3,5	B,C1,C2	K122E,I126T,P151H,L162R
D14	2.68E+01	Clone2,3	B,C2	K122E,I126T,M133T,F158L,L162R
D15	2.75E+01	Clone1,2,3,4	B	I110L,S113T,T118I,K122E,T143S,D144G,P151L
D16	2.27E+02	Clone1,2,3,4	B,C1,C2	K122E,I126T,G130V,C149R,F158L,L162R
D18	6.61E+01	Clone1,5	B	P111L,C124R
D19	2.89E+01	Clone1,4,5	B,C1	C121Y,K122E,I126T,T131S,L162R,V168D
E1	2.01E+01	Clone1,4,5	B	L104S,C147R,F158Y
F1	7.43E+06	/	B	No
F2	2.21E+06	/	C1	No
F3	3.56E+06	/	B	M133L
F4	4.53E+06	/	C1	I126V
F5	1.85E+06	/	B	No
F6	2.35E+06	/	B	P120S
F7	7.82E+06	/	B	M133L
F8	3.42E+06	/	C1	No
F9	5.82E+06	/	C1	No
F10	2.90E+06	/	B	No

### 3.3 Analysis of point mutations in the MHR region in the OBI and CHB groups

**Figure 1:**
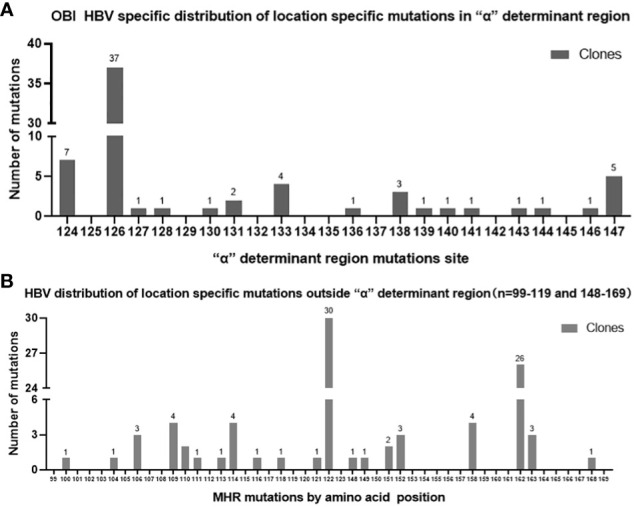
**(A)** OBI group “α” determinant Mutations site by different clones; **(B)** OBI group MHR Mutations site by different clones(outside”α” determinant).

In the OBI group, a total of 155 mutations occurred at 36 sites in the MHR region, of which 153 were amino acid substitutions and two were amino acid deletions, with the fifty different types of amino acid substitutions, with a total mutation rate of 3.21% (155/4828). The “α” determinant had the highest mutation frequency in the MHR region, with a total of 64 mutations and a mutation rate of 3.92% (64/1632). In the CHB group, a total of five mutations occurred at four sites in the MHR region, with a mutation rate of 0.70% (5/710), and a total of four mutations occurred at three sites in the “α” determinant, with a mutation rate of 1.67% (4/240). Mutation rate =Amino acid substitutions in MHR/”α” determinant region was divided by the total number of amino acid within the MHR/”α” determinant region of this sample(OBI/CHB group).The genetic mutation rate in the MHR region was significantly higher in the OBI group than in the CHB group, and the difference was statistically significant (P<0.01). (See **Table 3**) Sites with a higher number of point mutations in the MHR region (including the “α” determinant) were as follows (ranked according to the number of point mutations): s126 37 times(14/20 OBI and 1/10 CHB carried a mutation at s126, follow up as above), s122 30 times(14/20 OBI and 0/10 CHB), s162 26 times(14/20 OBI and 0/10 CHB), s124 7 times(5/20 OBI and 0/10 CHB), s147 5 times(2/20 OBI and 0/10 CHB), s109 4 times(3/20 OBI and 0/10 CHB), s114 4 times(3/20 OBI and 0/10 CHB),s133 4 times(3/20 OBI and 2/10 CHB), s158 4 times(3/20 OBI and 0/10 CHB), s106 3 times(2/20 OBI and 0/10 CHB), s138 3 times(1/20 OBI and 0/10 CHB), s152 3 times(3/20 OBI and 0/10 CHB), s163 3 times(2/20 OBI and 0/10 CHB), see **Figures 1A, B**.

**Table 3:** Analysis of the Mutation Rate in the MHR Region Concerning the B and C Genotypes.

Variable	Genotype status		P-value
	B(n=31)(%)	C(n=37)(%)	
K122E	1(3.2)	29(78.4)	<0.01
C124R	3(9.7)	4(10.8)	0.80
I126T	0	35(94.6)	<0.01
T126A	2(6.5)	0	0.20
T131A	1(3.2)	0	0.45
T131S	1(3.2)	0	0.45
M133T	1(3.2)	0	0.45
M133L	1(3.2)	1(2.7)	0.55
M133I	1(3.2)	0	0.45
C138R	2(6.5)	1(2.7)	0.87
T143S	1(3.2)	0	0.45
D144G	1(3.2)	0	0.45
C147R	1(3.2)	0	0.45
C147Y	3(9.7)	1(2.7)	0.48
L162R	0	26(70.3)	<0.01

### 3.4 Analysis of the common mutation types in the MHR region in genotype B and C

**Figure 2:**
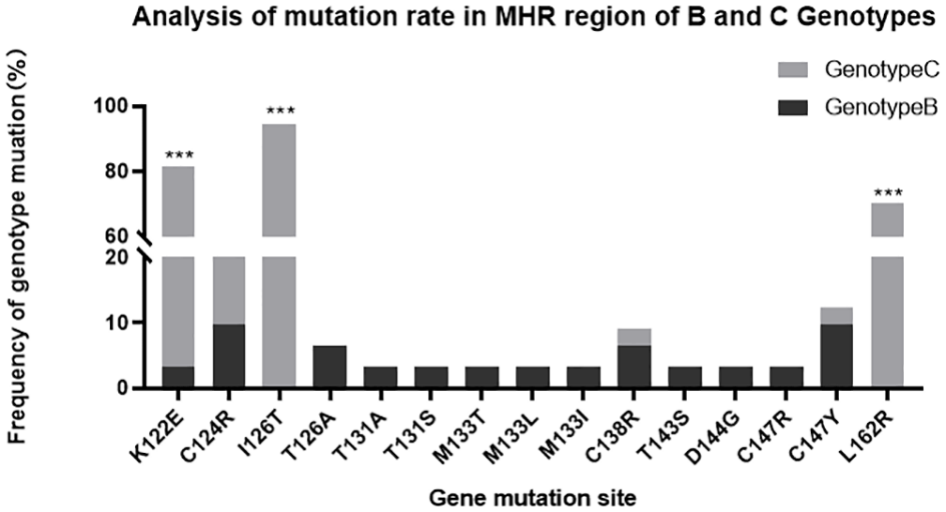
MHR region of B and C genotypes. ***P<;0.01, the difference was considered statistically significant.

Among the 100 clones in the OBI group, 68 cloned strains were confirmed by the sequencing results as S-region genes in HBV, of which 31 were type-B clones with 19 mutations, and 37 were type-C clones with 97 mutations. The number of type-C mutations was much higher than that of type-B mutations, and the difference was statistically significant (P<0.01). Among the many mutation sites in the MHR region, s126 had the highest mutation frequency, with a total of 37 mutations. Ats126, only two T126A mutations occurred in the B genotype, and all 35 mutations that occurred in the C genotype were I126T (P<0.01). The mutation site with the second highest mutation frequency was s122, with a total of 30 occurrences of K122E, one in the B genotype, and 29 in the C genotype (P<0.01). There were 26 mutations at s162, all of which being L162R in the C genotype (P<0.01). There were 7 mutations at s124, all of which being C124R, with three in the B genotype, and four in the C genotype (P>0.05). The genotype distribution of the other mutation sites are shown in **Figure 2** and **Table 3**.

## 4 Discussion

In this study, 42 samples which tested positive in HBV DNA were collected from the OBI group and amplified by two rounds of PCR reaction. Target gene bands were successfully amplified in only 20 cases, of which 12 cases had a HBV DNA load lower than 10^2^IU/ml. This was consistent with the existent literature which reports that the HBV DNA viral loads in most OBI samples are at low levels (Wang et al., 2023). In the OBI group, a total of 155 mutations occurred at 36 sites in the MHR region, with a total mutation rate of 3.21% (155/4828); the “α” determinant had the highest mutation frequency in the MHR region, with a total of 64 mutations and a mutation rate of 3.92% (64/1632). Huang X reported a significantly lower amino acid mutation rate of only 1.96% (42/2136) within the “α” determinant of OBI patients compared to the mutation rate observed in this region in our study (Huang et al., 2017). In the CHB group, a total of five mutations occurred at four sites in the MHR region, with a mutation rate of 0.70% (5/710), consistent with the mutation rate of 0.72% reported in the MHR region among CHB patients treated with nucleoside analogue-naïve, they observed a significant increase in the mutation rate to 1.31% following lamivudine treatment (Ding et al., 2014); a total of four mutations occurred at three sites in the “α” determinant, with a mutation rate of 1.67% (4/240). The genetic mutation rate in the MHR region was significantly higher in the OBI group than in the CHB group, and the difference was statistically significant (P<0.01). After comparing the sequencing results, the cloned strains from the 20 OBI samples were classified as B and C genotypes, which were in line with the epidemic pattern concerning HBV genotypes in our country. There were six cases of HBV type-B infection, five cases of type-C infection, and nine cases of mixed infection of type B and type C. Among them, two cases of mixed infection had type-C virus strains of two different serotypes. Nearly half of the OBI-infected patients had a history of previous HBV infection, resulting in mixed infection of different genotypes. The number of genetic mutations in the S-region of the type-C HBV strain was significantly higher than that of the type-B strain, especially with regard to the mutations in the “α” determinant. The mutations in this region can lead to changes in the antigenicity and immunogenicity of HBsAg, causing misdiagnosis and accelerating the progression of the disease. Further, studies have shown that patients infected with type-C HBV display a lower spontaneous clearance rate of HBsAg, a higher level of virus replication, faster disease progression, and poorer treatment efficacy than patients infected with type-B HBV (Ding et al., 2015).

s126 was the site with the highest mutation frequency in the “α” determinant, with a total of 37 mutations, including 35 I126T and two T126A, of which T126A only occurred in the B genotype, and I126T only occurred in the C genotype (P<0.01). Interestingly, s126 was T in type B and I in type C. There was an amino acid substitution with type B at site 126 in type C, and T126I was not found in type B. It has been reported that there are three amino acid forms at s126, namely s126I, s126T, and s126A. All types of amino acids at this site have comparable abilities in binding with HBsAb (Qiu et al., 2008), which also indicates that the new amino acid mutation sites generated by the two types of mutations I126T and T126A in this experiment did not affect the detection of HBsAg. Kim HS studied the mutation sites of the S-region gene covered by the Roche Elescsys HBsAg II assay kit and showed that neither the I126T nor the T126A mutations lead to missed detection of HBsAg, which is consistent with the above-mentioned literature (Kim et al., 2018). There is also literature showing that the antigenicity of HBsAg is significantly reduced in the case of I/T126S mutation: The virus replication was significantly reduced in both vivo and vitro experiments. This affects the secretion of virus particles, which may lead to immune escape, OBI, and vaccine escape in chronic HBV infection (Zhu et al., 2016).

s122 in the MHR region was also one of the high-frequency mutation sites, with a total of 30 mutations, second only to s126.All mutations were K122E, of which 29 cases occurred in type C, and only one case occurred in type B. This kind of mutation is associated with HBV subgenotypes (P<0.01). Studies have shown that sites 120 to 123 in the “α” determinant play a crucial role in the antigenicity of HBsAg. The K122E mutation has not been reported in the literature, while the K122I mutation has been studied more extensively. The mutant plasmid is transfected into Huh7, Hela, and other cells, and the antigenicity, immunogenicity, intracellular and supernatant HBsAg, virus replication and secretion were all inhibited to a certain extent (Huang et al., 2016). Little is known about the K122E mutation. Could this mutation affect HBsAg detection, and if yes, how? These questions need to be further explored.

There were 26 mutations at s162 in the MHR region, all of which were L162R in type C, but none was in type B. Genetic correlation was present (P<0.01). The mutations at s162 have not been reported in the literature, and s162 is located in the transmembrane structure (TM), which is currently less studied. TM is mainly composed of hydrophobic amino acids. The hydrophobic feature allows the amino acids to be inserted into the phospholipid bilayer and firmly rivets the S-protein to the outer membrane of the HBV, which is crucial for HBsAg to interact with the core particles, assemble mature virus particles, and secrete cells (Zhang et al., 2019; Jiang et al., 2022). Therefore, we speculate that the L162R mutation changes the more hydrophobic leucine into the extremely hydrophilic arginine, which may damage the hydrophobicity of the TM domain and produce important changes to the structure and function, or antigenicity, of the S-protein, thereby preventing the HBsAg from binding to the detection antibody, resulting in OBI. The OBI caused by the L162R mutation in the TM region has not been reported in the previous literature. Further research is required to determine whether the frequent missed detection of HBsAg is directly related to the damage in the hydrophobic structure of the TM.

A total of seven mutations occurred at s124, including three mutations in type B and four mutations in type C, all of which were C124R mutations. This makes s124 the site with the second highest mutation frequency in the “α” determinant, s126 being that with the highest. s147 had a total of five mutations, of which there was one C147R mutation in type B and four C147Y mutations in both type C (three times) and type B (once). The “α” determinant is rich in cysteine (C), and the connection between C124-C137 and C139-C147 forms a disulfide bond, maintaining the structure and antigenic conformation of the two loops (Li et al., 2017). When the C124 and C147 cysteines are replaced, the disulfide bond cannot be formed, and the loop structure is destroyed, which changes the antigenicity and immunogenicity of HBsAg, making it unable to be recognized by the HBsAg detection antibody, resulting in OBI (Shi et al., 2012). The s124 mutations which are known at present include C124R/Y/A. Studies have reported that C124R mutation reduces HBsAg antigenicity and virus particle secretion in Huh7 cells and mouse models (Huang et al., 2016). The s147 mutation commonly reported in the literature is C147A/R. C147A can severely impair the secretion of virions, and C147R affects the secretion of HBsAg and virions (Kwei et al., 2013). There is no literature about C147Y. Further research is needed to determine whether it is related to OBI.

s133 was another important mutation site in the “α” determinant and produced three different types of mutations, M133L, M133T, and M133I.It had a total of four mutations, among which only one M133L mutation was in type C, and the other three were all in type B. The three mutation types which occurred in this study had all been reported in the literature. Abundant literature shows that the M133L mutation is often associated with vaccine immune escape (Lazarevic, 2014). The M133T mutation can generate a new glycosylation site ^131^NST^133^, which interferes with HBsAg recognition by mimicking B cell epitopes and impairs virion secretion (Yu et al., 2014). No T131N mutation was found in this study. We only found a M133T single-site mutation without a new glycosylation site. Both Ito K and Kim HS found that M133T single-site mutations do not affect the secretory expression and antigenicity of HBsAg, and even appear frequently in HBsAg-positive samples (Ito et al., 2010; Kim et al., 2018). In addition, the M133T mutation can correct the decreased viral particle secretion caused by many mutation sites, such as I110M, G145R, N146Q, N146S, R169H, etc (Ito et al., 2010). In this study, the M133L mutation appeared both in the OBI group and in the CHB control group, whose samples had a high viral load and tested strongly positive in HBsAg. This suggests that M133L may not be the main factor causing the missed detection of HBsAg. The reason for this phenomenon calls for further exploration.

In general, the MHR mutation frequency in the OBI group was significantly higher than that in the CHB control group, and the difference was statistically significant. The missed detection of HBsAg was related to the amino acids caused by genetic mutations in the MHR. Most of the “hotspot” mutations in this study have been reported in previous literature. These mutations can affect the secretory expression, antigenicity and immunogenicity, as well as virion synthesis of HBsAg, resulting in missed detection of HBsAg, thus giving rise to OBI. The discovery of these high-frequency “hotspot” mutation sites can help develop new serological detection reagents, improve the sensitivity of detection, and reduce the missed detection of HBsAg. The mutations in type-C genes found in this study such as K122E and L162R have rarely been reported. Do mutations of this type have an impact on the antigenicity, immunogenicity, secretion and expression of HBsAg, and how do they affect the detection of HBsAg? Further exploration is needed to answer these questions.

## Data availability statement

The raw data supporting the conclusions of this article will be made available by the authors, without undue reservation.

## Ethics statement

The studies involving humans were approved by the Department of Clinical Laboratory, the Seventh People’s Hospital Affiliated to Shanghai University of Traditional Chinese Medicine. The studies were conducted in accordance with the local legislation and institutional requirements. Written informed consent for participation was not required from the participants or the participants’ legal guardians/next of kin because the serum was discarded after clinical testing.

## Author contributions

CH: Data curation, Formal analysis, Funding acquisition, Methodology, Writing – original draft, Writing – review & editing. YL: Data curation, Methodology, Writing – original draft, Writing – review & editing. XJ: Data curation, Formal analysis, Supervision, Writing – original draft. ZXu: Data curation, Formal analysis, Methodology, Writing – original draft. ZXi: Data curation, Formal analysis, Methodology, Writing – original draft. ZL: Formal analysis, Funding acquisition, Methodology, Supervision, Writing – original draft, Writing – review & editing.
